# Stretching Actin Filaments within Cells Enhances their Affinity for the Myosin II Motor Domain

**DOI:** 10.1371/journal.pone.0026200

**Published:** 2011-10-13

**Authors:** Taro Q. P. Uyeda, Yoshiaki Iwadate, Nobuhisa Umeki, Akira Nagasaki, Shigehiko Yumura

**Affiliations:** 1 Biomedical Research Institute, National Institute of Advanced Industrial Science and Technology (AIST), Tsukuba, Ibaraki, Japan; 2 Biomedicinal Information Research Center, National Institute of Advanced Industrial Science and Technology (AIST), Koto, Tokyo, Japan; 3 Department of Functional Molecular Biology, Graduate School of Medicine, Yamaguchi University, Yamaguchi, Yamaguchi, Japan; 4 Precursory Research for Embryonic Science and Technology (PRESTO), Japan Science and Technology Agency (JST), Kawaguchi, Saitama, Japan; Consejo Superior de Investigaciones Cientificas, Spain

## Abstract

To test the hypothesis that the myosin II motor domain (S1) preferentially binds to specific subsets of actin filaments *in vivo*, we expressed GFP-fused S1 with mutations that enhanced its affinity for actin in *Dictyostelium* cells. Consistent with the hypothesis, the GFP-S1 mutants were localized along specific portions of the cell cortex. Comparison with rhodamine-phalloidin staining in fixed cells demonstrated that the GFP-S1 probes preferentially bound to actin filaments in the rear cortex and cleavage furrows, where actin filaments are stretched by interaction with endogenous myosin II filaments. The GFP-S1 probes were similarly enriched in the cortex stretched passively by traction forces in the absence of myosin II or by external forces using a microcapillary. The preferential binding of GFP-S1 mutants to stretched actin filaments did not depend on cortexillin I or PTEN, two proteins previously implicated in the recruitment of myosin II filaments to stretched cortex. These results suggested that it is the stretching of the actin filaments itself that increases their affinity for the myosin II motor domain. In contrast, the GFP-fused myosin I motor domain did not localize to stretched actin filaments, which suggests different preferences of the motor domains for different structures of actin filaments play a role in distinct intracellular localizations of myosin I and II. We propose a scheme in which the stretching of actin filaments, the preferential binding of myosin II filaments to stretched actin filaments, and myosin II-dependent contraction form a positive feedback loop that contributes to the stabilization of cell polarity and to the responsiveness of the cells to external mechanical stimuli.

## Introduction

Actin filaments play a variety of important roles in eukaryotic cells, and each of their functions depends on a specific set of actin binding proteins. Indeed, it is generally believed that local regulation by actin binding proteins determines the function of the actin filaments in that area [1], [2]. In polarized amoeboid cells, for instance, Arp2/3-dependent polymerization of actin filaments pushes the membrane of the leading edge forward. At the same time, cofilin is enriched in the area slightly behind the leading edge, where it promotes the disassembly and turnover of the actin filaments. In the posterior of those cells, active interaction between actin filaments and bipolar myosin II filaments contracts the cortex, assisting detachment of the cell rear from the substrate and propulsion of the cytoplasm in a forward direction. Similarly, active interaction between actin filaments and myosin II filaments constricts the contractile rings in dividing cells.

Biochemical and biophysical studies of the interaction between actin filaments and various actin-binding proteins are providing insight into the mechanisms underlying the functional differentiation of actin filaments *in vivo*. Most importantly, it is now well established that actin filaments assume multiple conformations, depending upon the binding of nucleotides and/or actin binding proteins [3], [4], [5], [6]. It has also been shown that in certain cases the conformational changes are highly cooperative in the sense that the binding of an actin binding protein to an actin subunit within a filament induces conformational changes in neighboring subunits. For instance, the binding of cofilin changes the conformation within individual actin subunits as well as the interaction between the subunits, leading to significant shortening of the helical pitch [7], [8]. The binding of cofilin to actin filaments is highly cooperative, which leads to the formation of cofilin clusters along the filaments under certain conditions, and the changes in helical pitch induced by cofilin can extend well beyond the clusters into the bare zone of the filaments [8]. One way to interpret these observations is that cofilin binding induces cooperative conformational changes in neighboring actin subunits, which in turn increases the affinity of the neighboring actin subunits for cofilin [9], leading to cluster formation. A slightly different way of interpreting these observations is that actin subunits within filaments thermally fluctuate among multiple semi-stable structures, and cofilin binds to segments with a favorable structure, thereby stabilizing that structure [8]. This view is supported by the observations that pure actin filaments naturally have variable twist [10]. Egelman and his colleagues [11] went on to demonstrate that subunits within native actin filaments take one of the six distinct conformations, and subunits within a segment of the filament take the same conformation, representing strong cooperativity. Although questioned by another recent, high resolution electron microscopic analysis [12], we feel cooperative polymorphism of pure actin filaments plausible because it is able to explain well-established cooperative conformational changes of unbound subunits induced by binding of actin binding proteins to neighbor subunits within the same filament.

Studies of cooperative conformational changes to actin filaments induced by myosin have a longer history. For instance, skeletal heavy meromyosin (HMM)-induced increases in the signal from fluorescently labeled actin subunits saturate when the molar concentration of HMM is only 1/10 that of the actin subunits [13]. Similar saturating effects of HMM or its motor domain (subfragment 1 or S1) at significantly sub-stoichiometric concentrations have been observed using several different techniques [14], [15], [16], [17], [18]. Furthermore, the binding of HMM to actin filaments is cooperative *in vitro* [19], [20]. In the case of Ca^2+^-actin filaments in the absence of ATP, this cooperativity results in the clustering of HMM molecules in some parts of the filament, which leaves other parts of the filament bare [19]. In the case of physiological Mg^2+^-actin filaments in the presence of low concentrations of ATP, the cooperativity is weaker in that some of the actin filaments appear bare, while others are sparsely bound with HMM molecules [20]. This weaker cooperativity cannot be explained by direct interactions between HMM molecules because they are separated by unbound actin subunits; instead, it most likely involves cooperative conformational changes in the actin subunits that increase the affinity of neighboring actin subunits for HMM.

If this weaker cooperative binding between HMM and actin filaments reflects the preferential binding of HMM to subunits with a favorable conformation among multiple semi-stable conformations, as was suggested for the cooperative binding of cofilin to actin filaments [8], it would lead to an interesting hypothesis that the myosin II motor domain selectively binds to specific subsets of actin filaments having a favorable conformation, which would contribute to the proper intracellular localization of myosin II filaments *in vivo*. This view is apparently inconsistent with the observation that filament formation is necessary for proper intracellular localization of myosin II in *Dictyostelium* [21], [22] and *Drosophila* S2 cells [23], and that GFP-fused S1 of *Dictyostelium* myosin II is diffusely distributed in the cytoplasm (T. Uyeda, unpublished observation). We speculate that the myosin II motor domain has a stronger affinity for subsets of actin filaments with a favorable conformation, but detection of this preferential binding *in vivo* is difficult because the time-averaged affinity between the motor domain and the actin filaments in the presence of ATP is too weak in the absolute sense. In the present study, therefore, we expressed two GFP-fused S1 mutants with amino acid substitutions that enhanced its affinity for actin filaments in the presence of ATP. It was our expectation that these GFP-S1 mutants could serve as probes enabling detection of subsets of actin filaments having a higher affinity for the myosin II motor domain *in vivo*. The results demonstrate that these GFP-S1 mutants do indeed preferentially bind to subsets of actin filaments; more specifically, they bind to mechanically stretched subsets of the filaments *in vivo*. Here we present a novel scheme whereby stretch-induced changes in actin filament conformation and the resultant promotion of myosin II binding help amoeboid cells to stabilize front-to-rear polarity and to respond to external mechanical stimuli.

## Materials and Methods

### Cell culture and expression of fluorescently labeled proteins

Wild-type *Dictyostelium discoideum* AX2 cells and mutant cells lacking *mhcA* (encoding myosin II heavy chain), *ctxA* (encoding cortexillin I) or *pten* (encoding PTEN) were grown in plastic Petri dishes containing HL-5 medium [24] supplemented with penicillin and streptomycin at 22°C. Cells were transfected by electroporation with the *Dictyostelium* expression vector pBIG [25], pTIKL [26], pDdNeo or pDdBsr (Fig. S1) harboring a gene encoding a GFP- or mCherry-fusion protein. Transfectants were selected and grown in HL-5 medium containing 12 µg/ml G418 and/or 10 µg/ml blasticidin S.

The construction of the plasmids to express fluorescently labeled proteins is detailed in Text S1.

### Live cell observation using confocal fluorescence microscopy

Live cell imaging was accomplished in the following two ways. Cells expressing GFP-mutant S1 or GFP-myosin II heavy chain were settled on plastic Petri dishes with thin glass bottoms (Iwaki Glass, Japan) and observed using an Olympus IX-70 microscope equipped with a PlanApo 100× (NA = 1.35) oil-immersed objective and a confocal laser scanning unit (CSU 10, Yokogawa, Japan). To obtain chemotactic cells, the cells were starved for 8–10 h in 17 mM K^+^-Na^+^-phosphate buffer (pH 6.4) before imaging. To image cells undergoing cytokinesis C, the cells were incubated for 3 days in HL-5 medium containing 12 µg/mL G418 in a Teflon flask on a rotating shaker and then allowed to settle onto a glass-bottomed dish for 15 min. The medium was then replaced with K^+^-Na^+^-phosphate buffer, and the cells were imaged as above.

To observe flattened cells live, the cells were overlaid with a thin agarose sheet, as described previously [27].

### Observation of fixed cells using confocal fluorescence microscopy

Cells on glass-bottomed dishes were simultaneously permeabilized and fixed by replacing the K^+^-Na^+^-phosphate buffer with a solution containing 10 mM Pipes (pH 6.8), 3 mM MgCl_2_, 1 mM EGTA, 1 mM DTT, 0.1% Triton X-100, and 1% glutaraldehyde. After fixing the cells for 10 min, they were stained for 1 h in PBS containing 3 nM rhodamine-phalloidin (Rh-Ph), rinsed in PBS containing 10 mM DTT, and observed using the IX-70 confocal microscope. Superimposition of two pseudocolored images (GFP and rhodamine) of the same cells was accomplished using ImageJ software (http://rsb.info.nih.gov/ij/).

Alternatively, cells flattened with an agarose sheet were fixed in ethanol containing 1% formalin. They were then stained with Rh-Ph after washing with PBS, and observed using a confocal microscope (LSM510 Meta, Carl Zeiss) equipped with a 100× Plan Neofluor objective (NA = 1.3). Argon (488 nm line) and HeNe (543 nm line) lasers were used for excitation of GFP and rhodamine, respectively. Ratiometric images were calculated from the GFP and rhodamine images of the same cells using Image Calculator in ImageJ.

### Aspiration assays

Portions of cells co-expressing GFP-mutant S1 and mCherry-actin were aspirated into a pipette as described previously [28]. Briefly, a suction pipette with an inner diameter of 3 µm was made from a glass capillary (G-1, Narishige, Tokyo, Japan) using a pipette puller (PG-1, Narishige) and a microforge (MF-830, Narishige). The pipette was then connected to a vertical open-ended glass tube and a 5 ml syringe via a silicone tube, and all three were filled with Bonner's salt solution (10 mM NaCl, 10 mM KCl, 3 mM CaCl_2_). The syringe was then used to adjust the height of the water surface in the glass tube so that the hydrostatic pressure at the mouth of the suction pipette is 2.5 kPa. The cells were observed using the LSM510 confocal microscope.

## Results

### Probes used in this study

To identify subsets of actin filaments with a higher affinity for the myosin II motor domain, we needed two types of probes: one that would accurately report local concentrations of total actin filaments and another that would preferentially bind to subsets of actin filaments having a higher affinity for the myosin II motor domain.

To observe actin filaments within cells using fluorescence microscopy, three distinct classes of probes were available: GFP-actin [29], [30], [31], GFP-actin binding domain (ABD) of actin binding proteins (e.g., GFP-Lifeact) [32], and Rh-Ph. Staining patterns of GFP-actin, GFP-Lifeact and Rh-Ph were compared in fixed and permeabilized *Dictyostelium* cells, which demonstrated that those of GFP-actin and Rh-Ph were more similar to one another than those of GFP-Lifeact and Rh-Ph were (Text S2 and Fig. S2). Thus, we chose to stain cells with Rh-Ph after fixation and permeablization, in order to detect total actin filaments in a semiquantitative manner.

Visualization of actin filaments having increased affinity for the myosin II motor domain required the use of a fluorescently labeled motor domain lacking the tail domain, since filament formation involving the tail domain is able to localize myosin II filaments *in vivo* (reviewed by [33]). However, GFP-fused myosin II S1 appeared to be always diffusely distributed in the cytoplasm (movie S1). This was presumably because in the presence of ATP, myosin II S1 spends most of its time in the ATPase cycle carrying ADP and phosphate, and associates only weakly with actin filaments. Slow, actin-stimulated release of phosphate from the S1-ADP-Pi complex establishes strong binding to the actin filament, followed by a rapid power stroke and ADP release. In the presence of physiological concentrations of ATP, rebinding of the nucleotide is rapid, and S1-ATP almost immediately dissociates from actin filaments, so that the time spent strongly bound to the actin is relatively short [34], [35], which makes the time-averaged affinity of S1 for actin in the presence of ATP very low. Several S1 mutations that enhance its affinity for actin in the presence of ATP have been reported. In *Dictyostelium*, G680A myosin II S1 exhibits very slow actin-stimulated ADP release, which extends the strongly bound state and increases its time-averaged affinity for actin in the presence of ATP [36], [37]. The corresponding G699A mutant skeletal myosin II also exhibits strong affinity for actin in the presence of ATP [38]. L596S S1 exhibits a very high affinity for actin in the weakly bound state and accelerated transition to the strongly bound state, again resulting in a higher time-averaged affinity for actin in the presence of ATP. The mechanism by which the L596S mutation increases the affinity of S1 for actin in the weakly bound state is unknown, but it is presumably allosteric, since Leu596 is not situated on myosin's actin binding face [39]. We incorporated the G680A or L596S mutation into S1 fused N-terminally to GFP and C-terminally to a FLAG tag. When expressed in wild-type *Dictyostelium* cells, each of the two GFP-S1 mutants showed a distinct cortical localization, and was particularly abundant along the front and rear cortex in polarized cells (movie S2). This pattern of localization was similar to that reported for actin filaments, which was consistent with the enhanced affinity of the S1 mutants for actin filaments in the presence of ATP. We therefore decided to use these GFP-S1 mutants as probes for actin filaments having increased affinity for the myosin II motor domain.

### Comparison of the patterns of GFP-S1 mutant localization and Rh-Ph staining

**Figure 1:**
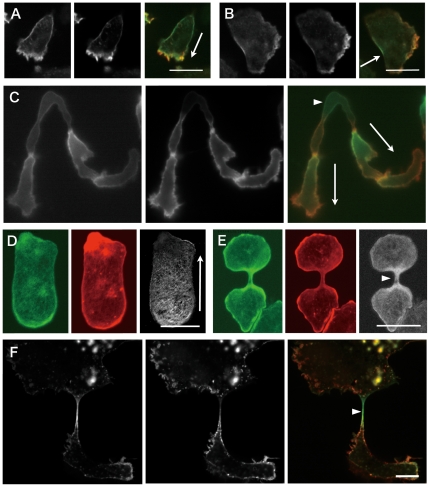
Relative signal intensities from localized Rh-Ph and myosin II GFP-S1. Cells expressing GFP-L596S S1 were permeabilized/fixed and stained with Rh-Ph. A: A starved and polarized wild-type cell. The arrow shows the direction of movement, and the left, middle and right images of this and panels B, C and F are GFP fluorescence image, rhodamine fluorescence image, and superimposition of the two pseudocolored images. B: A starved wild-type cell moving in a keratocyte-manner [31]. C: Starved and streaming wild-type cells. The cell indicated by the arrowhead is bi-axial, with both ends of the cell advancing. D: Similar to A, except that this cell was flattened by a sheet of agarose and the right panel shows a GFP/Rh ratiometric image. E: A dividing wild-type cell under an agarose sheet, with a ratiometric image on the right. The arrowhead shows the cleavage furrow. F: A large, multinucleate myosin II-null cell undergoing cytokinesis C. The arrowhead shows the cytoplasmic strand. Bars: 10 µm.

To semi-quantitatively compare the local abundances of the GFP-S1 mutants and actin filaments *in vivo*, cells expressing a GFP-S1 mutant were simultaneously permeabilized and fixed in the presence of Triton X-100 and glutaraldehyde, then stained with Rh-Ph and observed under a confocal fluorescence microscope. At first glance, the distributions of Rh-Ph and GFP-L596S S1 fluorescence appeared similar in all of the cells examined. However, more careful comparison clearly demonstrated that there were distinct and reproducible differences between the two distributions. Superimposition of pseudocolored GFP and rhodamine images of the same cells showed that Rh-Ph fluorescence was stronger along the front edge and along filopod-like thin projections, whereas the GFP fluorescence was stronger along the sides and the rear cortex in polarized cells (Fig. 1A, B). A similar pattern was prominent in chemotactically streaming cells during the development phase (Fig. 1C). Because the distributions of GFP-G680A S1 and GFP-L596S S1 were very similar (supplemental Fig. S3A), we mainly used GFP-L596S S1 as the probe for actin filaments with a higher affinity for the myosin II motor domain in subsequent experiments.

The relative intensities of the Rh-Ph and GFP fluorescence signals were also visualized through ratiometric representation, which clearly showed the GFP signal divided by the rhodamine signal to be stronger along the sides of the cell and in the rear cortex (Fig. 1D). In a dividing cell, GFP fluorescence was enriched in both the equatorial and polar regions (movie S3), but ratiometric images showed that the GFP signal was relatively stronger in the equatorial region than the polar regions (Fig. 1E).

In contrast, GFP fluorescence was distributed mainly in the cytoplasm when cells expressing GFP-wild-type S1 were processed in the same manner, and it was difficult to compare the relative intensities of the Rh-Ph and GFP fluorescences between different parts of the cortex because of the weakness of the GFP signals (supplemental Fig. S3B).

These results suggested that actin filaments along the sides and rear cortices in polarized cells and the equatorial cortices in dividing cells had higher affinities for GFP-L596S S1 than actin in other areas. These higher affinity actin filaments are typically bound to endogenous myosin II filaments [27], [40], and were thus presumed to be mechanically stretched. This led us to speculate that conformational changes in actin filaments induced by either mechanical stretching or biochemical changes related to the recruitment of myosin II enhanced the affinity of the filaments for GFP-L596S S1. One might speculate that GFP-S1 probes bound to myosin II or to some other actin binding protein on actin filaments, rather than directly to the actin filaments. This possibility was unlikely, however, because robust binding of the GFP-S1 probes to actin filaments *in vivo* required either of the two mutations that enhanced the affinity of the probe for purified actin filaments in the presence of ATP *in vitro*. Furthermore, to experimentally rule out the possibility that the GFP-S1 probes bound to specific actin filaments through direct interaction with myosin II, the localization of GFP-L596S S1 was characterized in myosin II-null cells. These cells are unable to divide in suspension culture and so become very large and highly multinucleate after 3 days. When subsequently placed on a substrate, they quickly adhere and different parts of the large cells move in different directions. Eventually, a thin cytoplasmic strand is formed between each cell fragment and the rest of the cell, which is severed after further pulling by the movement of the cell fragment, effectively resulting in cell cycle-uncoupled cell division (cytokinesis C or traction-mediated cytofission) [41], [42], [43]. During this process, the cytoplasmic strands are greatly stretched in an apparently passive manner, without myosin II. Live confocal imaging revealed that GFP-L596S S1 fluorescence was significantly enriched along the cortex of the cytoplasmic strands during this stretching (movie S4 and arrows in Supplemental Fig. S4). It was also noted that GFP-L596S S1 was enriched along the retracting cortex in those myosin II-null cells (movie S4 and arrowheads in Supplemental Fig. S4). Double labeling of permeabilized, fixed cells and superimposition of pseudocolored GFP and rhodamine images of the same cell demonstrated that, although Rh-Ph fluorescence was enriched along the cortex of the stretched cytoplasmic strands, the enrichment of GFP-L596S S1 in that area was far more pronounced (Fig. 1F). Thus, the enhanced binding of the GFP-L596S S1 to a subset of actin filaments reflects conformational changes in the actin filaments, rather than direct interaction with endogenous myosin II.

To test the possibility that the C-terminal FLAG tag or the two light chain binding domains with the light chains bound were involved in the localization of GFP-L596S S1, another mutant, GFP-L596S S1ΔIQ, which lacked both the light chain binding domains and the FLAG tag, was expressed in wild-type cells. GFP fluorescence from this chimeric protein was also enriched along the sides and the posterior of polarized cells, and along the cytoplasmic strands during cytokinesis C (Supplemental Fig. S3C, D). Based on these observations, we conclude that the GFP-S1 probes recognized the conformation of a subset of actin filaments with enhanced affinity for the myosin II motor domain.

### GFP-fused myosin I motor domain

Although myosin II is normally localized along the sides and posterior of polarized cells, other classes of myosin show different intracellular distributions. Most notably, myosin I (myoB and myoD) localizes along the leading edges of polarized *Dictyostelium* cells [44]. This prompted us to ask whether the myosin I motor domain prefers to bind to the same subset of actin filaments as the myosin II motor domain. To address that question, we initially expressed a GFP-fused myoB motor domain lacking the light chain binding domain (myoB-S1ΔIQ). However, after we failed to detect significant intracellular localization of the GFP fluorescence in a preliminary experiment, two point mutations expected to increase the protein's affinity for actin in the presence of ATP were introduced (GFP-S332D/G607A myoB-S1ΔIQ). S332D is an activating mutation at the so-called “TEDS rule” site [45]. Given that Gly607 of myoB corresponds to Gly680 of myosin II, and this Gly residue between the so-called SH1-SH2 helices is absolutely conserved among diverse myosins, we presumed that G607A likely increases the affinity of myosin I for ADP and, hence, its time-averaged affinity for actin in the presence of ATP. Although we have no biochemical data as to the consequences of those two mutations, the combination resulted in more pronounced localization of GFP-myoB-S1ΔIQ along the cortex and in the leading pseudopods (Fig. S5).

**Figure 2:**
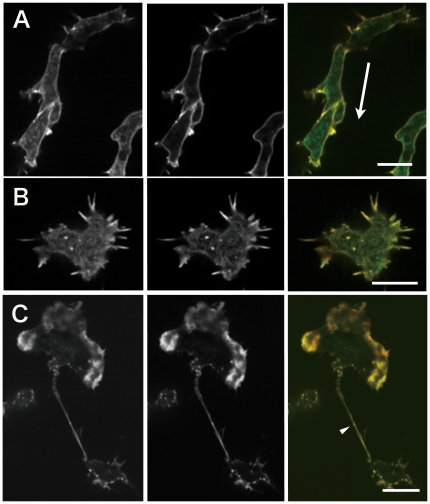
Relative signal intensities from localized Rh-Ph and GFP-myosin I motor domain. Cells expressing GFP-S332D/G607A myoB-S1ΔIQ were permeabilized/fixed and stained with Rh-Ph. A: Starved and streaming wild-type cells. The arrow shows the direction of movement. B: A myosin II-null cell with numerous filopodia. C: A large, multinucleate myosin II-null cell undergoing cytokinesis C. The left, middle and right image of each triplet is a GFP fluorescence image, rhodamine fluorescence image and superimposition of the two pseudocolored images. Bars: 10 µm.

In wild-type cells that were permeabilized, fixed and stained as above, GFP-S332D/G607A myoB-S1ΔIQ was found mainly in the cytoplasm, distributed in a punctate manner, but was also localized along the cortical actin filaments and in the filopodia (Fig. 2A, B). However, superimposition of pseudocolored GFP and rhodamine images of the same cell shows that, unlike GFP-L596S S1, GFP-S332D/G607A myoB-S1ΔIQ was not concentrated along specific subsets of actin filaments. Likewise, GFP-S332D/G607A myoB-S1ΔIQ was not enriched along the cytoplasmic strands to a greater degree than Rh-Ph during cytokinesis C in myosin II-null cells (Fig. 2C).

### Response to aspiration-induced cortical stretching

**Figure 3:**
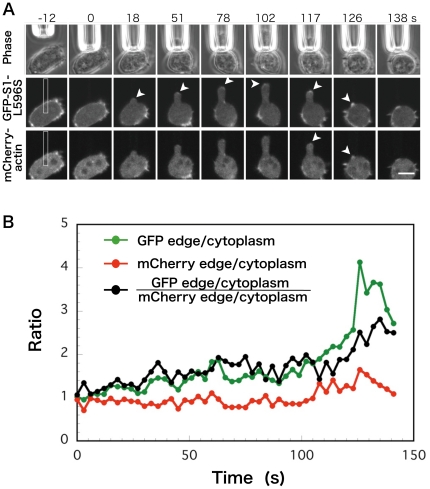
Relocalization of GFP-L596S S1 and mCherry-actin in wild-type cells in response to local aspiration using a microcapillary. A: Accumulation of GFP-L596S S1 and mCherry-actin at the cortex in the portions of the cell deformed by aspiration (arrowheads). The numbers indicate time after initiation of suction. Bar: 10 µm. This result is representative of 8 experiments. B: Time sequence showing the accumulation of GFP-L596S S1 and mCherry-actin at the cortex in the aspirated portion of the cell shown in panel A. Fluorescence intensities in the cytoplasmic area and hemispherical tip area of the aspirated portion in the rectangle were quantified, and the edge/cytoplasm ratios were calculated for each fluorescence image. Accumulation of GFP fluorescence (green line) is evident at 15 s, while increase in mCherry fluorescence (red line) is detectable only after 105 s. Overall, the edge/cytoplasm ratio was higher for GFP than mCherry (black line), and there was no strong correlation between the accumulations of GFP and mCherry fluorescence in this or any other sequences (not shown).

We next tested whether GFP-L596S S1 would also preferentially bind to cortical actin filaments when the cell cortex was stretched due to an external stimulus. For this experiment, we used a microcapillary to apply negative pressure to the cell cortex. We and others previously showed that myosin II transiently accumulates along the cortex when it is sucked into a capillary, and suggested that myosin II-dependent cortical contraction then contributes to the escape of the cell from the capillary [28], [46], [47]. Live fluorescence imaging showed that GFP-S1-L596S was also enriched along the cortex near the tip of the area drawn into the microcapillary (movie S5 and Fig. 3A). Dual color live imaging of wild-type cells co-expressing mCherry-actin and GFP-L596S S1 showed that actin also accumulates along the aspirated cortex (movie S5 and Fig. 3A), but detailed comparison of the time-dependent changes in the fluorescence profile revealed that there was a poor correlation between the accumulation of GFP-L596S S1 and mCherry-actin, and that accumulation of GFP-L596S S1 usually preceded that of mCherry-actin (Fig. 3B). These results indicated that at least the initial increase in GFP-L596S S1 was not dependent on an increase in actin filaments, which was consistent with the idea that GFP-L596S S1 preferentially binds to stretched actin filaments, whether the stretch is due to the cell's own force or to an externally applied force.

In parallel experiments, GFP-S332D/G607A myoB-S1ΔIQ expressed in wild-type cells was mostly cytoplasmic, and we were unable to detect distinct localization along the cortex inside or outside the aspirated areas of live cells (movie S6).

### Effects of knocking out genes known to affect myosin II localization

**Figure 4:**
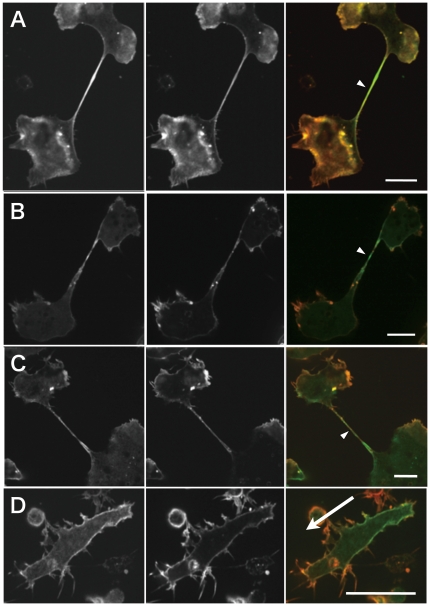
Relative signal intensities of localized Rh-Ph and GFP-L596S S1 (A, B, D) or GFP-myosin II (C) in various knockout cells. Knockout cells lacking PTEN (*pten*-) or cortexillin I (*ctxA*-) and expressing GFP-L596S S1or GFP-myosin II heavy chain were permeabilized/fixed and stained with Rh-Ph. The left, middle and right panel of each triplet is a GFP fluorescence image, rhodamine fluorescence image, and superimposition of the two pseudocolored images. A: Large, multinucleate *pten*- cell undergoing cytokinesis C. B and C: Large, multinucleate *ctxA*- cells undergoing cytokinesis C. D: A starved and streaming *ctxA*- cell. The arrow shows the direction of movement. Bars: 10 µm.

Knocking out *pten* [28] or *ctxA* [48] gene in *Dictyostelium* impairs stretch-induced local accumulation of myosin II *in vivo*. To explore the possible involvement of their products, PTEN and cortexillin I, respectively, in the preferential binding of the myosin II motor domain to stretched actin filaments *in vivo*, we investigated the behavior of GFP-L596S S1 in *pten*- and *ctxA*- cells. Like myosin II-null (*mhcA*-) cells, *pten*- cells failed to divide efficiently and became multinucleate during 3 days in suspension culture [49], and then underwent typical cytokinesis C on glass substrates. In those cells, GFP-L596S S1 accumulated extensively along the cytoplasmic strands, as in myosin II-null cells (Fig. 4A). *ctxA*- cells also frequently failed to divide in suspension culture [50] and then underwent cytokinesis C on glass substrates; and again GFP-L596S S1 accumulated along the cytoplasmic strands (Fig. 4B). Starved and chemotactically streaming *ctxA*- cells appeared different from wild-type cells in that the mutants had numerous protrusions along their sides. Nonetheless, the fluorescence from the GFP-L596S S1 was relatively stronger in the rear cortex in these cells than the Rh-Ph fluorescence (Fig. 4D). These results indicated that neither PTEN nor cortexillin I plays an essential role in the preferential binding of GFP-L596S S1 to stretched actin filaments *in vivo*.

Finally, we expressed GFP-myosin II heavy chain in *ctxA*- and *pten*- cells induced to undergo cytokinesis C as above, and found that in both cases the GFP-myosin II accumulated along the cytoplasmic strands during cytokinesis C (Fig. 4C and movie S7).

## Discussion

### Mechanism of preferential binding of the myosin II motor domain to stretched actin filaments

Non-muscle myosin II transiently forms bipolar filaments and associates with specific subsets of actin filaments to drive local contraction of the cell cortex. This leads to a number of important cellular activities, including contraction of contractile rings and retraction of the rear of polarized cells. To fulfill those functions, myosin II filaments must selectively bind to appropriate subsets of actin filaments within the cell, and three different mechanisms have been suggested to play roles in this process in *Dictyostelium* and other model cells. These are, local assembly/disassembly of myosin II filaments [33], [51], [52], directional transport of myosin II filaments riding on the flow of cortical actin filaments [53], [54], [55], and association of the backbone of myosin II filaments with one or more components of the cell cortex [23], [56], [57], [58], [59]. Notably all three of these mechanisms require myosin II to be in the filament state, a notion that is supported by the observation that assembly-incompetent mutant myosin II is unable to localize in *Dictyostelium* [21], [22] or *Drosophila* S2 cells [23]. Here, we demonstrated another mechanism, in which individual myosin II motor domains or S1 molecules preferentially bind to mechanically stretched subsets of actin filaments. Use of L596S or G680A S1 mutant was necessary because the time-averaged affinity of wild-type S1 for actin filaments was too weak in the presence of ATP to detect distinct intracellular localizations (movie S1 and Fig. S3). We believe that the localizations we observed with the GFP- S1 mutants reflect the intrinsic properties of the myosin II motor domain, as the two mutations appear to enhance the affinity for actin filaments through different molecular mechanisms. Consistent with this premise, GFP-fused S1 of non-muscle myosin IIB was shown to bind more strongly to stress fibers than to peripheral actin filaments in normal rat kidney cells [60]. Similarly, the myosin II motor domain of fission yeast (Myo2p) is reportedly enriched along contractile rings [58]. Intriguingly, within each of these cell types stress fibers and contractile rings are composed of mechanically stretched actin filaments, which suggests that preferential binding to mechanically stretched subsets of actin filaments is a common property of the myosin II motor domain, except that the actin affinity of the motor domain of rat and yeast myosin II in the presence of ATP is relatively stronger than that of *Dictyostelium*'s.

**Figure 5:**
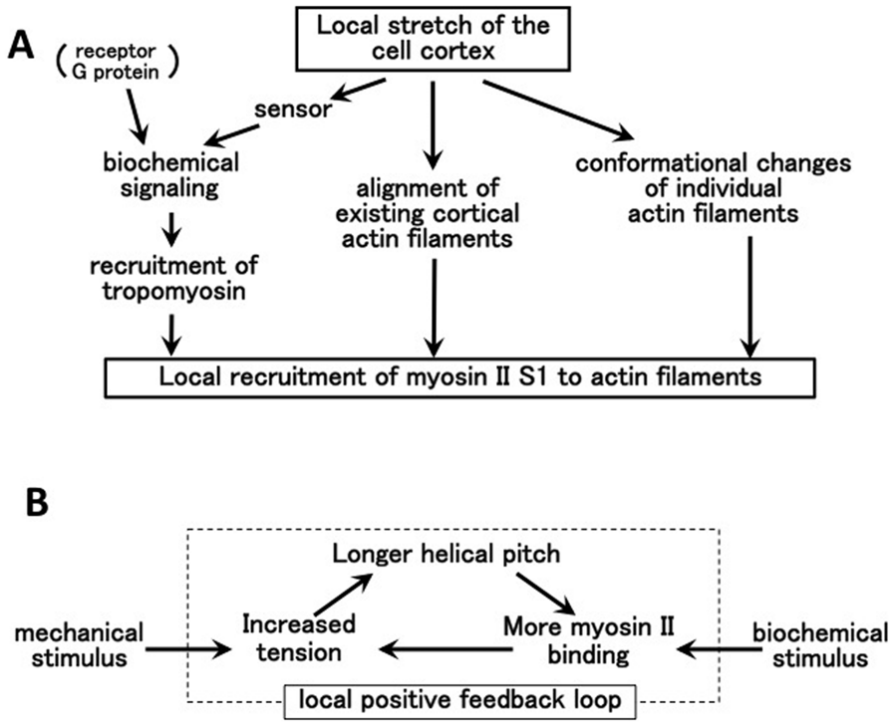
A: Three molecular mechanisms for recruiting myosin II S1 to stretched actin filaments *in vivo*. B: Possible physiological function of a three-component positive feedback loop consisting of stretch-induced conformational changes to actin filaments, preferential binding of the myosin II filaments to stretched actin filaments, and myosin II-dependent tension generation.

Three different molecular mechanisms might contribute to the enhanced binding of the myosin II motor domain to stretched actin filaments (Fig. 5A). A conventional view would assume a mechanosensor that triggers a biochemical pathway that ultimately leads to enhanced affinity of actin filaments for myosin II. For instance, it has been shown that tropomyosin isoforms differentially regulate the affinities of actin filaments for different classes of myosin motors [61], [62], [63]. According to Tang and Ostap [60], this differential regulation explains the enhanced binding of the rat non-muscle myosin IIB motor domain, but not that of the myosin I motor domain, to stress fibers along which tropomyosin is enriched. However, *bona fide* tropomyosin genes have not been identified in the completely sequenced *Dictyostelium* genome, making it difficult to speculate that biochemical signaling involving tropomyosin plays an important role in the stretch-induced recruitment of myosin II motors to actin filaments in *Dictyostelium* cells. In addition, although PTEN and cortexillin I have been implicated in the recruitment of myosin II filaments to stretched cortex in *Dictyostelium* [28], [48], we found that neither of those molecules is required for the preferential binding of GFP-L596S S1 to stretched actin filaments.

The second mechanism assumes stretch-induced higher order structural changes to the actin cytoskeleton. In the relaxed cell cortex, individual actin filaments are oriented more or less randomly [64], but mechanical stretching of the cortex would align the filaments in the direction of the stretch. The myosin II motor domain – *e.g.*, the proteolytic muscle S1 [65] or recombinant His tagged *Dictyostelium* S1 (T. Uyeda, unpublished data) – tends to form bundles of actin filaments in the absence of ATP *in vitro*. It is thus possible that a GFP-S1 mutant carrying a mutation that increases its affinity for actin in the presence of ATP prefers to bind to the aligned actin filaments enriched in the stretched areas. On the other hand, the GFP-S1 mutants were not enriched in filopodia, which contain parallel bundles of actin filaments, as they were in the rear cortex of polarized cells. It is unlikely this is due to some unfavorable geometry of the filaments within the bundles, such as parallel *vs.* anti-parallel alignments, because exogenously-added GFP-S1 efficiently bound to filopodial actin filaments in Triton X-100-treated cells (S. Yumura, unpublished data). Moreover, GFP-S332D/G607A myoB-S1ΔIQ bound to similar degrees along filopodial actin bundles and cortical actin filaments (Fig. 2), ruling out the possibility that limited accessibility prevented the binding of the myosin II GFP-S1 mutants to the filopodial actin bundles.

The third mechanism assumes that stretching induces structural changes in individual actin filaments at the atomic level. It is well established that actin filaments are able to assume multiple conformational states, in which individual actin subunits take on different structures (for review, see [5], [11]). A number of actin binding proteins [6], [66], [67], [68], [69], [70], including skeletal muscle myosin II [71] and brain myosin V [72], have been shown to change the structure of actin filaments. Those structural changes most likely increase the affinity of the filaments for that particular actin binding protein (cooperative binding), as has been demonstrated in the case of cofilin [8], [73], [74]. In addition, those conformational changes to the filament could modulate the affinity for other actin binding proteins, either positively or negatively [3] - *e.g.*, so that the preferential binding of a specific group of actin binding proteins to a particular subset of actin conformers would lead to the exclusion of other actin binding proteins [75]. At present, there is no detailed information about stretch-induced conformational changes to actin subunits within filaments, but molecular dynamics simulations suggest that mechanical stretching of actin filaments with a physiologically relevant force (200 pN) is able to untwist the helix and change the mechanical properties of the filament [76]. Furthermore, Shimozawa and Ishiwata detected a fluorescence increase when they stretched tetramethylrhodamine-labeled actin filaments, signaling the occurrence of stretch-induced changes in the atomic structure of the actin subunits [77]. The functional relevance of these conformational changes were confirmed by Sokabe and his colleagues, who found that cofilin severs stretched actin filaments more inefficiently than relaxed ones *in vitro* [78], suggesting actin subunits within stretched filaments may assume a conformation having a lower affinity for cofilin.

Intriguingly, cofilin binding not only changes the atomic structure of each subunit, but also reduces the helical pitch of the filaments (super twisting) [7], [8]. Conversely, the binding of skeletal S1 slightly untwists the helix of actin filaments [71] (T. Yasunaga, personal communication), raising the possibility that the myosin II motor domain prefers to bind to untwisted actin filaments. As mechanical stretching is suggested to untwist the actin filaments [76], therefore, it is highly plausible that S1 prefers to bind to stretched actin filaments. On the other hand, a population of pure actin filaments exhibits a spectrum of helical pitches in the absence of external forces [8], [10]. Thus, even in the absence of an applied force, a certain fraction of actin filaments will presumably possess a more untwisted conformation with a higher affinity for the myosin II motor domain; stretching induced by an applied force only increases the untwisted fraction.

For these reasons, and because we previously demonstrated the cooperative binding of myosin II to Mg^2+^-actin filaments without additional proteins or alignment of the filaments *in vitro* [20], we favor the third mechanism, in which stretch-induced changes in the atomic structure of actin filaments and/or untwisting of the helix attract the myosin II motor domain. However, we do not exclude the possible contributions of either or both of the other two mechanisms. Needless to say, the aforementioned regulatory mechanisms involving the assembly of myosin II filaments also play important roles in the intracellular localization of myosin II.

Interestingly, the myosin I motor domain did not preferentially bind to stretched actin filaments. This is again in line with the report from Tang and Ostap, who showed that GFP-fused myo1b, a rat myosin I, localized along the cell periphery but not along stress fibers [60]. Distinct intracellular localizations of members of the same family of actin binding proteins have been reported for calponin-homology proteins [79], coronin [80], tropomyosin [81] and talin [82]. Notably, distinct intracellular localizations along specific actin-containing structures were also observed with the GFP-fused, isolated ABDs of *Dictyostelium* α-actinin and filamin, both of which are calponin homology proteins [79]. This suggests that subtle differences in the actin binding face of homologous actin binding domains can result in preferential binding to different conformations of actin subunits. In this scenario, the filamin ABD, which binds to cortical actin cytoskeleton but not to those in protruding pseudopods [79], may share a similar preference for actin structures with the myosin II motor domain.

### Physiological relevance of the preferential binding of myosin II motor domain to stretched actin filaments

In *Dictyostelium*, myosin II filaments interact with actin filaments located at the rear of polarized cells, at the tips of retracting pseudopods, and along the contractile rings in dividing cells, and drive local contraction [27], [40]. Thus, if individual myosin II motor domains have a higher affinity for stretched actin filaments, that would lead to formation of a local positive feedback loop, consisting of accumulation of myosin II filaments, increased tension, and conformational changes within the actin filaments that attract additional myosin II filaments (Fig. 5B). The affinity between individual motor domains and actin filaments is too weak for stable association in the presence of ATP, which necessitated the use of S1 mutants in this study. However, myosin II filaments are able to stably associate with actin filaments in the presence of ATP because they contain large numbers of motor domains.

It was suggested that stretching actin filaments *in vitro* reduces their affinity for cofilin [78]. Thus, stretching actin filaments would attract myosin II and repel cofilin. Conversely, along the leading edges of polarized cells, polymerization of actin filaments pushing against the cell membrane may axially compress the filaments, and prevent the binding of myosin II while attracting cofilin. This is consistent with the anterior localization of cofilin in polarized *Dictyostelium* cells [83] and fish keratocytes [84], which would further super-twist the actin filaments in the anterior region, forming another local positive feedback loop. Those two local positive feedback loops would contribute to the stabilization of cell polarity established by other biochemical stimuli.

Additionally, the responsiveness of actin filaments to mechanical stretch would enable cells to respond to external mechanical stimuli or perturbations. Our aspiration experiments directly demonstrated such a possibility, in that the locally stretched portion of the cell cortex exhibited locally enhanced contractility, which enabled the cell to escape from the mechanical stimulus. When a portion of an unpolarized, round fragment of a fish keratocyte was pushed with the tip of a microneedle, the cell fragment gained front-rear polarity and started to move unidirectionally away from the microneedle [85]. Again, it may be that local deformation and stretching of the cell cortex enhanced the contractility at the site of deformation through recruitment of myosin II filaments, and made that portion of the cell the rear.

One key unanswered question in modern cell biology is how different actin filaments within the same cell interact with different binding proteins and perform different functions. Two nonexclusive mechanisms have been proposed [75]. One is that the nucleators of actin polymerization “imprint” the structure of the resultant filament, which specifies the binding partner and, consequently, the function of the filament. The fact that the binding of one gelsolin molecule at the barbed end of a filament affects the structure of the filament over a long distance [86] implies that such an imprinting mechanism is highly plausible. The other mechanism depends on the mutually inhibitory binding of two actin binding proteins to actin filaments, coupled with long-range cooperative conformational changes to the filaments. More specifically, it was recently shown that actin filaments in fission yeast cells bind either fimbrin or tropomyosin [87]. This mutually exclusive binding of fimbrin or tropomyosin appears to depend on the ability of fimbrin to inhibit tropomyosin binding, and the long-range cooperativity of actin filaments ensures that neighboring subunits within a filament take the same conformation status. Here, we suggest that there is a third mechanism that is not exclusive with respect to the two mechanisms summarized above: mechanical stretch-induced long-range cooperative conformational changes to actin filaments.

Finally, it is worth mentioning the extensibility of the thin filaments in skeletal muscle. Mechanical and X-ray diffraction measurements have established that active contraction stretches the thin filaments, which is accompanied by untwisting of the helix [88]. More recently, Tsaturyan *et al.* revealed that rigor binding of myosin heads, without significant tension, untwists the helix of thin filaments by ∼0.2%, and applied tension further stretches the helix by a similar amount [71]. Although muscle is a complex and highly ordered system and interpretation of these results needs caution, S1-induced untwisting of actin filaments was observed *in vitro* as well (T. Yasunaga, personal communication). This implies that, at least in skeletal muscle, thin filaments are extensible springs, albeit rather stiff ones. Moreover, with the reasonable assumption that skeletal myosin heads possess a higher affinity for untwisted actin filaments, since the binding of skeletal myosin heads untwists the helix, it is further suggested that a positive feedback loop similar to what we proposed in Fig. 5B is formed in skeletal muscle.

### Conclusions

Mechanical sensing and downstream signaling involving the cytoskeleton play important roles in cellular responses in both the short term and over long periods. A number of proteins involved in regulating the cytoskeleton [89], [90], [91], [92], as well as the myosin motor [93], [94], have been shown to possess mechanical sensitivity. In the present study, however, we suggest a new possibility, that actin filaments are themselves mechanical sensors, which further emphasizes the functional importance of the structural polymorphism of actin filaments [11].

## Supporting Information

Figure S1 pDdNeo. The gene to be expressed in the form GFP-fusion protein is subcloned between the *Bam*HI and *Sac*I sites. Truncated *Ddp*I is a 2,033 bp *Hin*dIII fragment of pBIG. pDdBsr carries a blasticidin S resistance cassette in place of G418 resistance cassette.
(TIF)

Figure S2 Comparison of the fluorescent probes for actin filaments. A: A wild-type *Dictyostelium* cell expressing GFP-Lifeact was permeabilized and fixed with 0.1% Triton X100 and 1% glutaraldehyde, stained with Rh-Ph, and observed using a confocal fluorescence microscope. B: A wild-type *Dictyostelium* cell expressing GFP-actin was permeabilized/fixed, stained with Rh-Ph, and observed as above. The left, middle and right panel in each triplet show a GFP fluorescence image, rhodamine fluorescence image, and superimposition of the two pseudocolored images. Arrows show the direction of movement. Bars: 10 µm.
(TIF)

Figure S3 Comparison of GFP and rhodamine fluorescence intensities in wild-type cells expressing GFP-G680A S1 (A) and GFP-wild-type S1 (B) after permeabilization/fixation and staining with Rh-Ph. Live cells expressing GFP-wild-type S1 were brightly fluorescent (movie S1), but most of the fluorescence was lost during the permeabilization/fixation procedure, presumably because most of the GFP-wild-type S1 molecules were not bound to actin filaments in the cells. Therefore the original GFP fluorescence image in B was very dark and needed brightness enhancement for visualization. C: Starved and streaming wild-type cells expressing GFP-L596S S1ΔIQ observed as above. D: GFP-L596S S1ΔIQ -expressing myosin II-null cell grown in suspension for 3 days and then allowed to undergo cytokinesis C on a glass substrate was observed as above. The left, middle and right panel in each triplet shows a GFP fluorescence image, rhodamine fluorescence image, and superimposition of the two pseudocolored images. Bars: 10 µm.
(TIF)

Figure S4 Montage sequence of movie S7. Accumulations of GFP-L596S S1 along cytoplasmic strands during cytokinesis C and along the retracting cortices are marked by arrows and arrowheads, respectively. Numbers show elapsed time in min. Bar: 20 µm.
(TIF)

Figure S5 Localization of wild-type and mutant GFP-myoB-S1ΔIQ. Wild type cells expressing GFP- wild-type myoB-S1ΔIQ (A) or GFP-S332D/G607A myoB-S1ΔIQ (B) were observed by confocal microscopy. GFP- wild-type myoB-S1ΔIQ was mostly diffuse in the cytoplasm and only weakly concentrated in the extending pseudopods (arrowheads). GFP-S332D/G607A myoB-S1ΔIQ was more prominently localized along the cell cortex (arrow) and in the extending pseudopods (arrowheads). Bar: 10 µm.
(TIF)

Movie S1 Starved wild-type cells expressing GFP-wild type S1. The width of this field is 85 µm, and the speed is 105×.
(MOV)

Movie S2 Starved wild -type cells expressing GFP-L596S S1. The width of this field is 85 µm, and the speed is 105×.
(MOV)

Movie S3 A wild-type cell expressing GFP-L596S S1 during cytokinesis under an agarose sheet. The width of this field is 33 µm, and the speed is 42×.
(MOV)

Movie S4 Myosin-null cells expressing GFP-L596S S1 undergoing cytokinesis C and retractions. Accumulations of GFP-L596S S1 along cytoplasmic strands during cytokinesis C and along the retracting cortices are marked in the montage sequence of this movie (Supplemental Fig. S4). The width of this field is 135 µm, and the speed is 420×.
(MOV)

Movie S5 Relocalization of GFP-L596S S1 and mCherry-actin in wild-type cells in response to local aspiration using a microcapillary. Speed: 50×. This is the data set shown in Fig. 3A.
(AVI)

Movie S6 Relocalization of GFP-S332D/G697A myoB-S1ΔIQ in wild-type cells in response to local aspiration using a microcapillary. Speed: 50×. This result is representative of 13 experiments.
(AVI)

Movie S7 Accumulation of GFP-myosin II along a cytoplasmic strand during cytokinesis C of a multinucleate *pten*- cell. The width of this field is 55 µm, and the speed is 35×.
(MOV)

Text S1 Construction of the plasmids to express fluorescently labeled proteins.
(DOC)

Text S2 Comparison of the fluorescent probes for actin filaments.
(DOC)
