# Supplementary figures and images for: Stretching Actin Filaments within Cells Enhances their Affinity for the Myosin II Motor Domain
Source: PLoS One. 2011 Oct 13;6(10):e26200. doi: 10.1371/journal.pone.0026200 (PMC3192770; doi:10.1371/journal.pone.0026200)

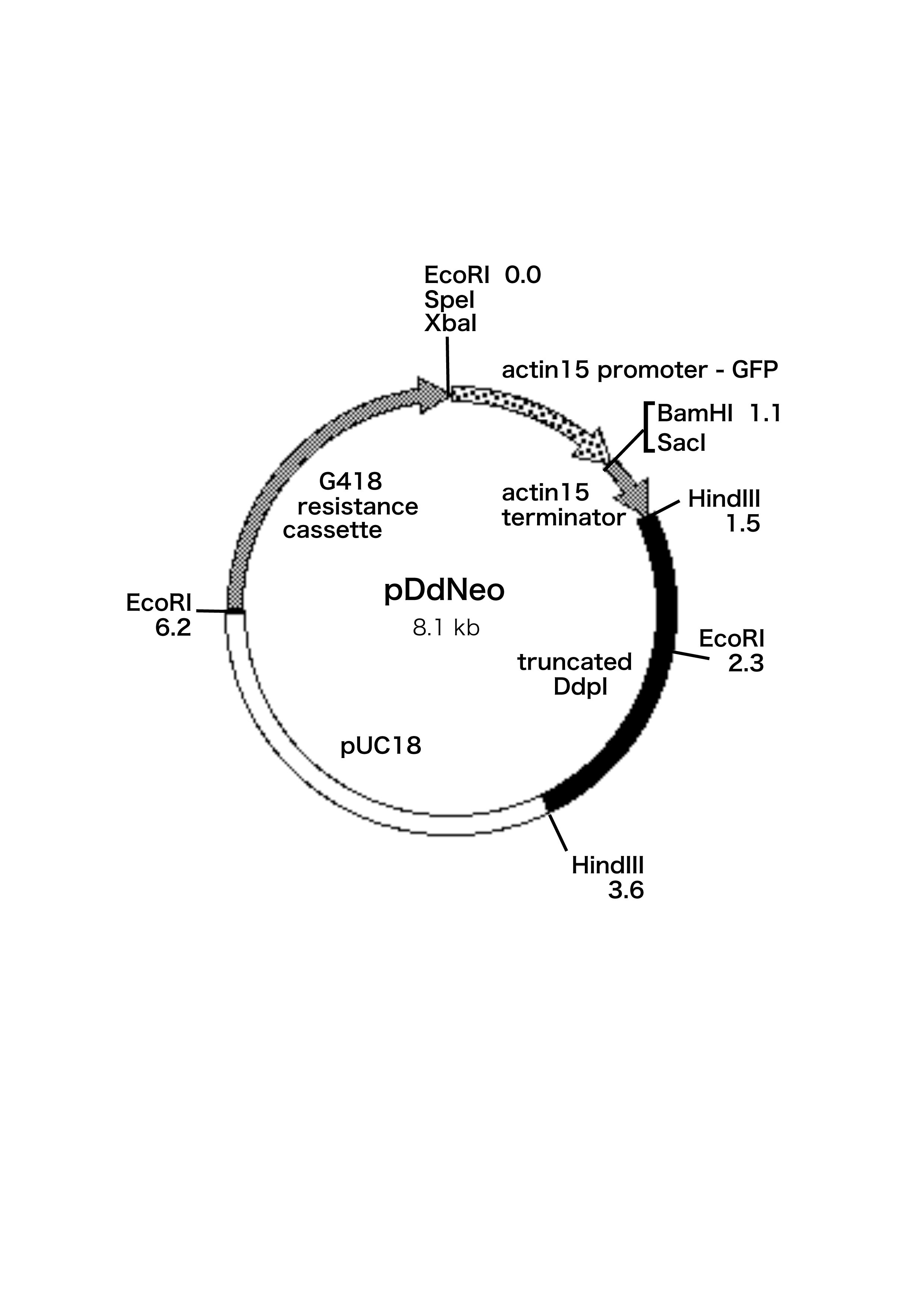

Supplement: Figure S1 — pDdNeo. The gene to be expressed in the form GFP-fusion protein is subcloned between the BamHI and SacI sites. Truncated DdpI is a 2,033 bp HindIII fragment of pBIG. pDdBsr carries a blasticidin S resistance cassette in place of G418 resistance cassette. (TIF) [file pone.0026200.s001.tif]

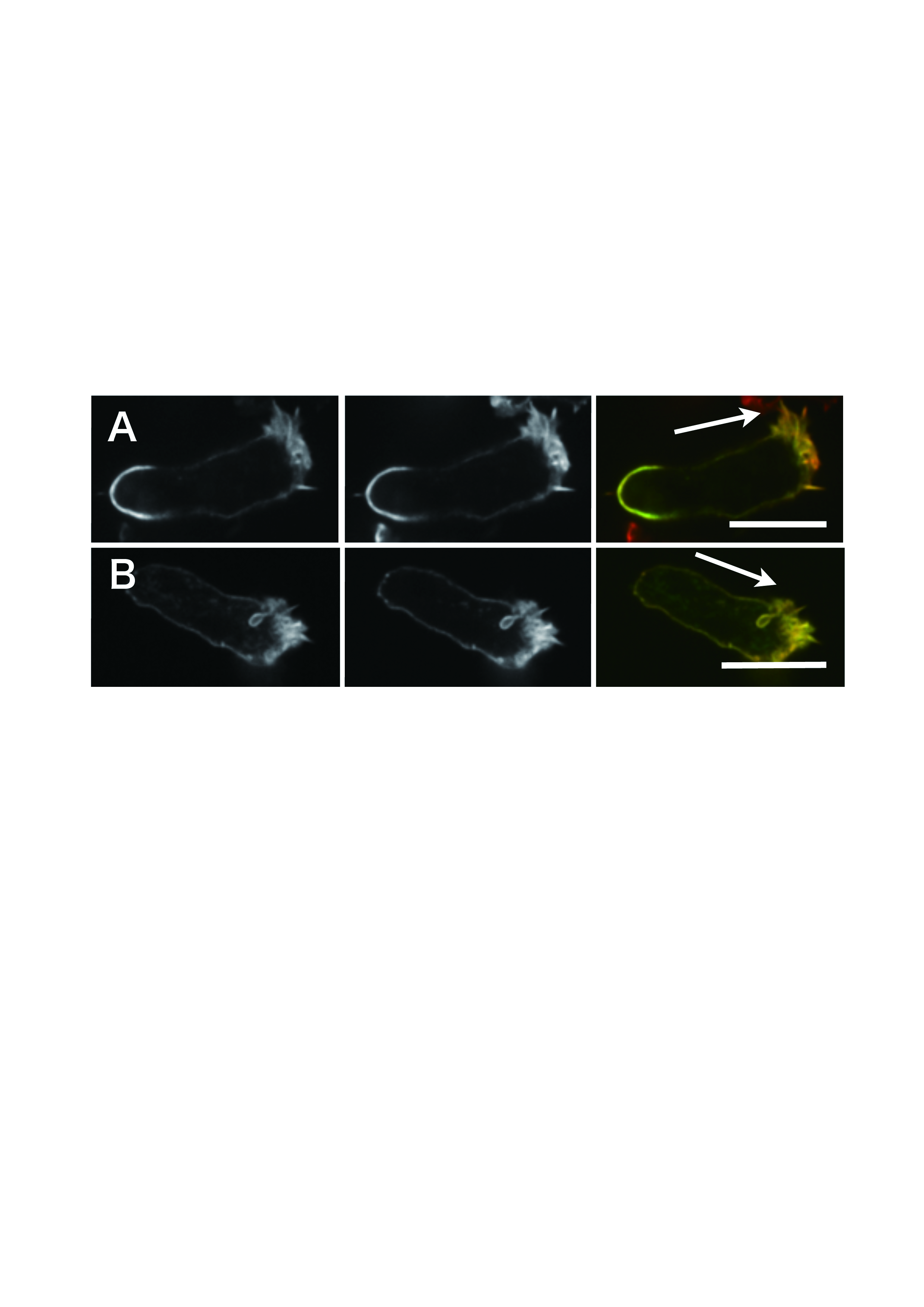

Supplement: Figure S2 — Comparison of the fluorescent probes for actin filaments. A: A wild-type Dictyostelium cell expressing GFP-Lifeact was permeabilized and fixed with 0.1% Triton X100 and 1% glutaraldehyde, stained with Rh-Ph, and observed using a confocal fluorescence microscope. B: A wild-type Dictyostelium cell expressing GFP-actin was permeabilized/fixed, stained with Rh-Ph, and observed as above. The left, middle and right panel in each triplet show a GFP fluorescence image, rhodamine fluorescence image, and superimposition of the two pseudocolored images. Arrows show the direction of movement. Bars: 10 µm. (TIF) [file pone.0026200.s002.tif]

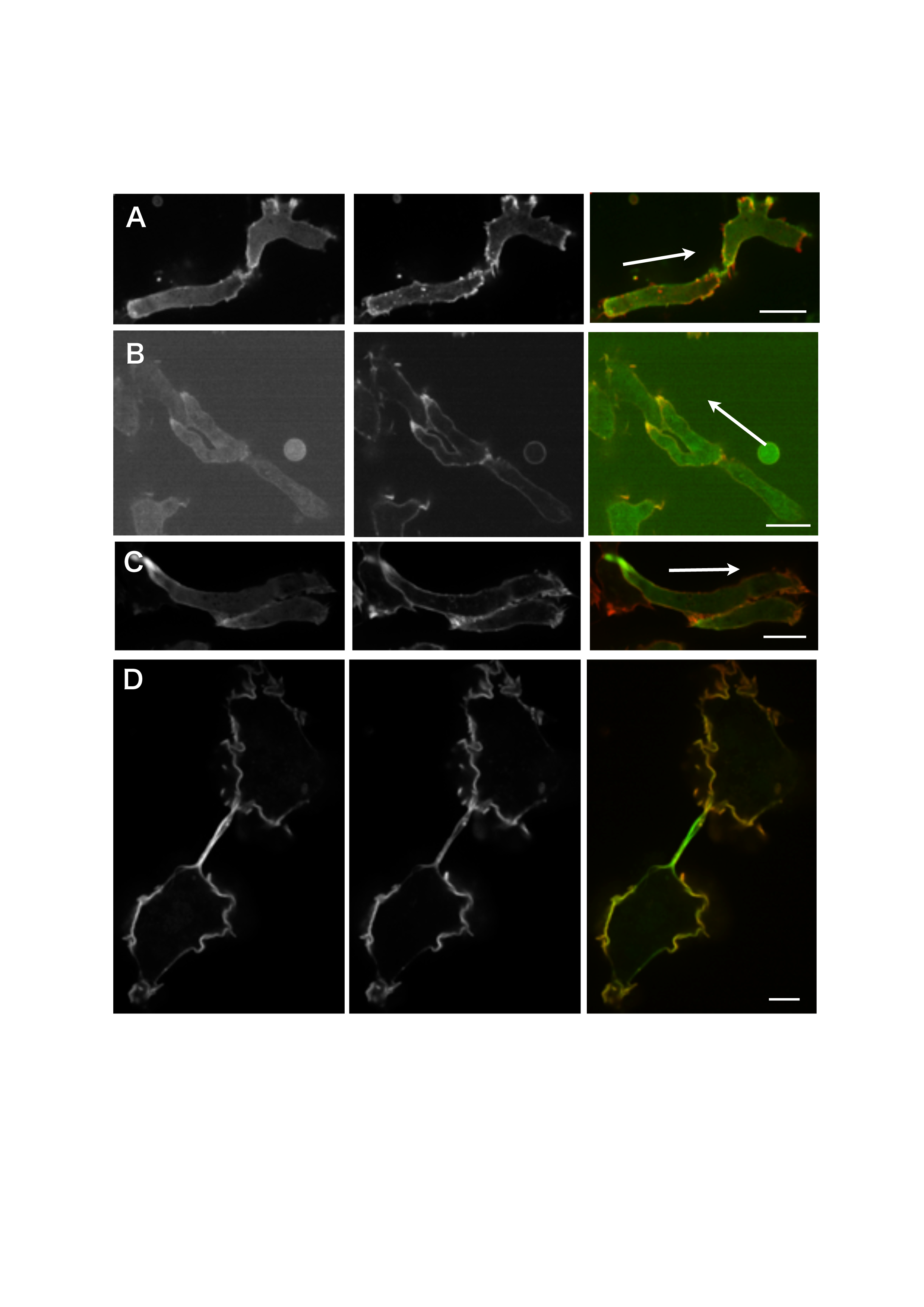

Supplement: Figure S3 — Comparison of GFP and rhodamine fluorescence intensities in wild-type cells expressing GFP-G680A S1 (A) and GFP-wild-type S1 (B) after permeabilization/fixation and staining with Rh-Ph. Live cells expressing GFP-wild-type S1 were brightly fluorescent (movie S1), but most of the fluorescence was lost during the permeabilization/fixation procedure, presumably because most of the GFP-wild-type S1 molecules were not bound to actin filaments in the cells. Therefore the original GFP fluorescence image in B was very dark and needed brightness enhancement for visualization. C: Starved and streaming wild-type cells expressing GFP-L596S S1ΔIQ observed as above. D: GFP-L596S S1ΔIQ -expressing myosin II-null cell grown in suspension for 3 days and then allowed to undergo cytokinesis C on a glass substrate was observed as above. The left, middle and right panel in each triplet shows a GFP fluorescence image, rhodamine fluorescence image, and superimposition of the two pseudocolored images. Bars: 10 µm. (TIF) [file pone.0026200.s003.tif]

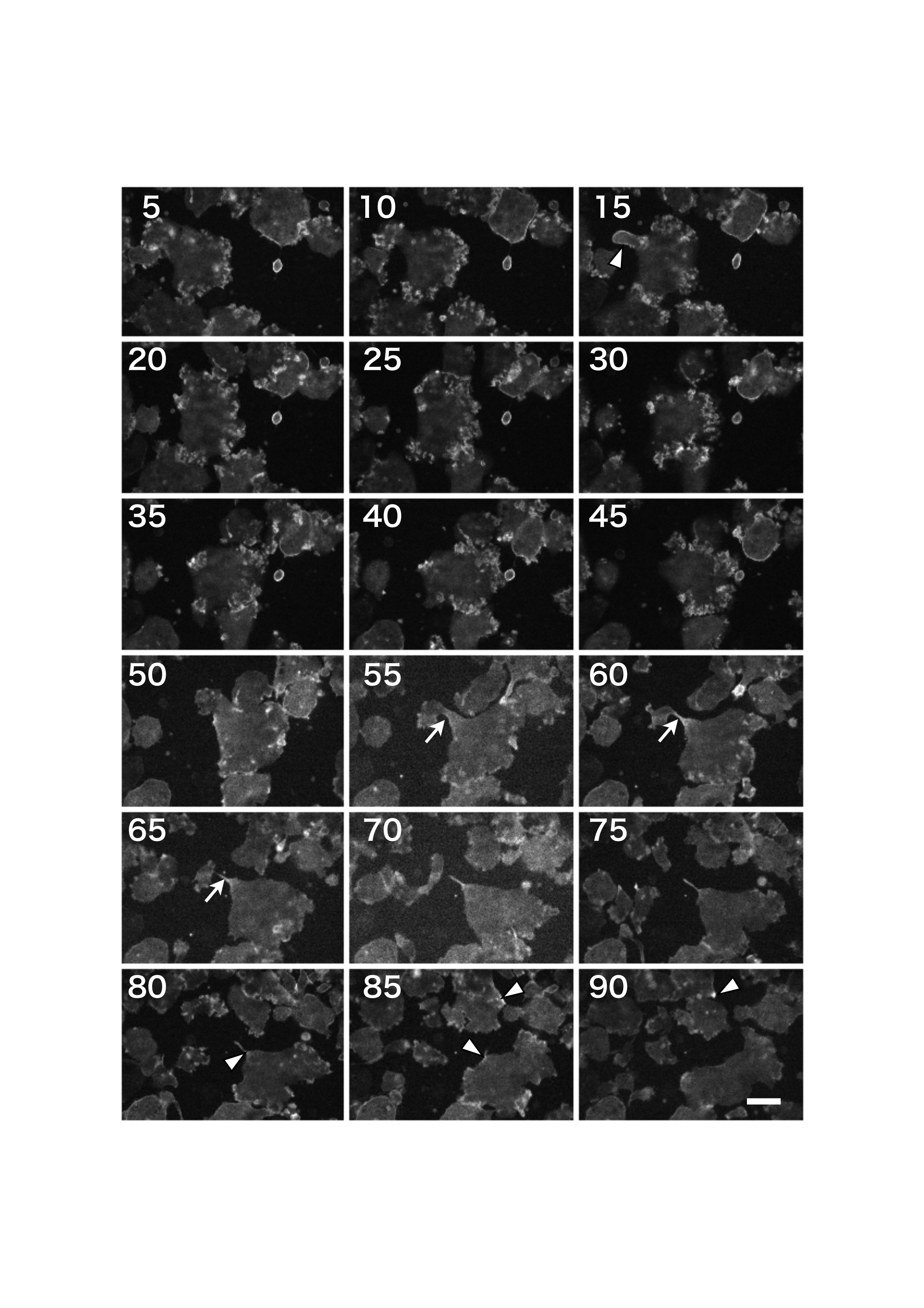

Supplement: Figure S4 — Montage sequence of movie S7. Accumulations of GFP-L596S S1 along cytoplasmic strands during cytokinesis C and along the retracting cortices are marked by arrows and arrowheads, respectively. Numbers show elapsed time in min. Bar: 20 µm. (TIF) [file pone.0026200.s004.tif]

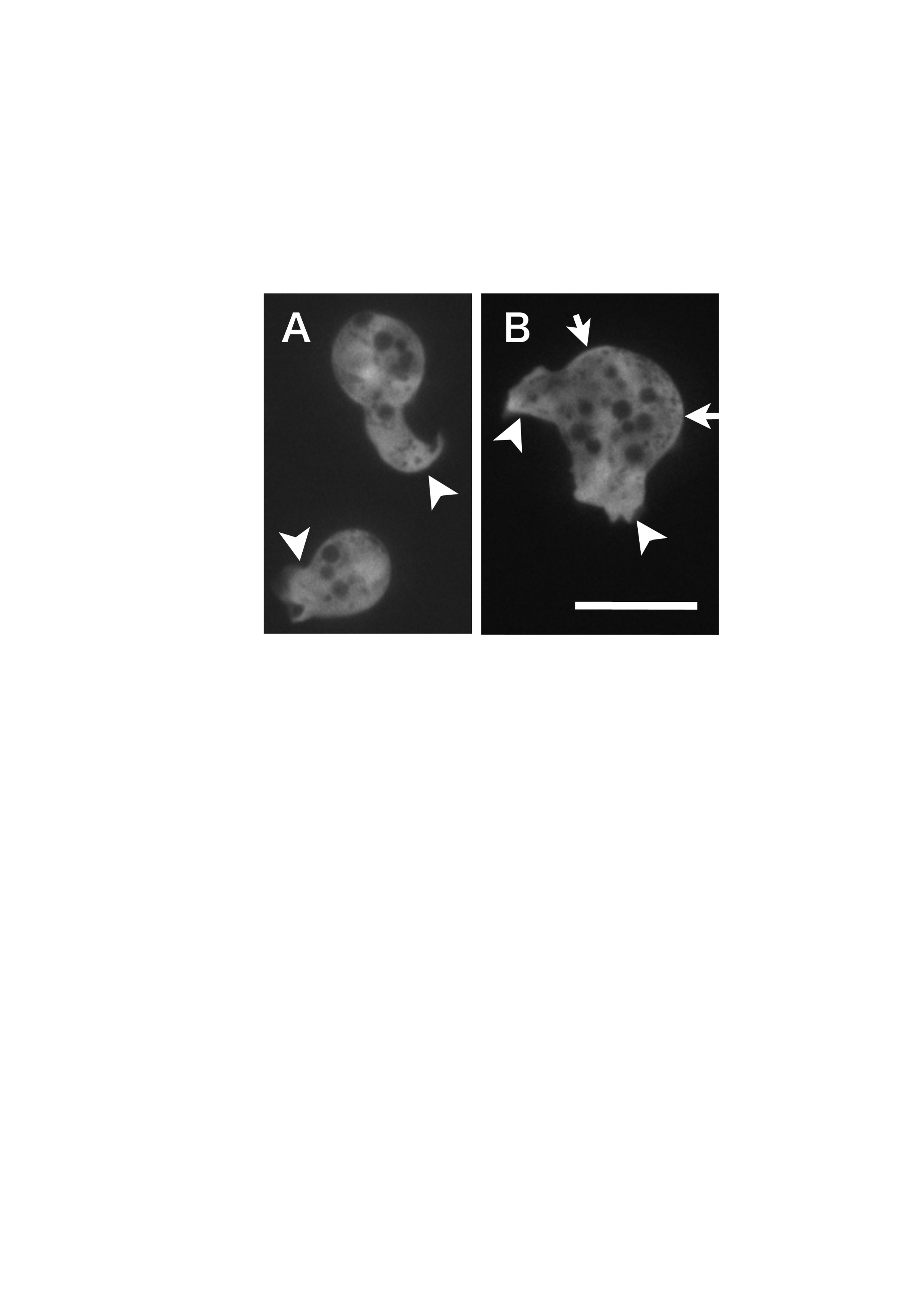

Supplement: Figure S5 — Localization of wild-type and mutant GFP-myoB-S1ΔIQ. Wild type cells expressing GFP- wild-type myoB-S1ΔIQ (A) or GFP-S332D/G607A myoB-S1ΔIQ (B) were observed by confocal microscopy. GFP- wild-type myoB-S1ΔIQ was mostly diffuse in the cytoplasm and only weakly concentrated in the extending pseudopods (arrowheads). GFP-S332D/G607A myoB-S1ΔIQ was more prominently localized along the cell cortex (arrow) and in the extending pseudopods (arrowheads). Bar: 10 µm. (TIF) [file pone.0026200.s005.tif]
