# Supplementary material for: Stretching Actin Filaments within Cells Enhances their Affinity for the Myosin II Motor Domain
Source: PLoS One. 2011 Oct 13;6(10):e26200. doi: 10.1371/journal.pone.0026200 (PMC3192770; doi:10.1371/journal.pone.0026200)
Supplement: Text S1 — Construction of the plasmids to express fluorescently labeled proteins. (DOC) [file pone.0026200.s013.doc]

Construction of plasmids

Shuttle vectors. Four shuttle vectors, pBIG [1], pTIKL, pDdNeo, and pDdBsr, were used in this study. pTIKL [2] is a derivative of pBIG , and consists of pBluescript (Stratagene) and a 7.1 kb fragment of pATNB43 [3] containing a G418 resistance cassette and the *Ddp*I sequence that enables extrachromosomal replication in *Dictyostelium discoideum*. However, because the sequence derived from pATNB43 contained sequences unnecessary for extrachromosomal replication, a miniaturized version, pDdNeo, was constructed by combining fragments of pTIKL (Supplemental Figure 1A). The size of pDdNeo, excluding the actin 15 promoter-GFP-actin 15 terminator cassette, was reduced to 6.6 kb, from 10.0 kb of pTIKL. In addition, to enable simultaneous expression of two proteins, a pDdBsr, pDdNeo-based plasmid with a blasticidin resistance cassette in place of G418 resistance cassette, was constructed (not shown).

GFP-myosin II: pTIKLGFPS65Tmyo [4] was used to express GFP-fused heavy chain of *Dictyostelium* myosin II. pTIKL GFPS65T-myo was derived from pBIG GFPmyo [5] by replacing the vector backbone from pBIG to pTIKL and including a S65T mutation in GFP.

GFP-S1: The sequence downstream of the unique *Nco*I site in *mhcA* of pTIKL GFPS65T-myo, which codes the tail domain, was replaced by 5’-CCATGGTGGAAACTCTTCTCAAAGGCTCCGTCCATTATTAAAGAGAAGAAACCCTCGAGGAGACTATAAAGATGACGATGACAAAGCTTAA to yield pTIKL GFP-S1. 　This C-terminal amino acid sequence of the resultant S1 was PWWKLFSKARPLLKRRNPRGDYKDDDDKA*. pTIKL GFP-L596S S1 and pTIKL GFP-G680A S1 were constructed by restriction enzymes-dependent conventional cloning strategies using pTIKL GFP-S1 and plasmids that express L596S S1 [6] or G680A myosin [7].

GFP-L596S S1∆IQ: The 3’-terminal portion of the S1 gene was modified by a PCR based method to 5’-ATTGAAGAAGCTCGTGAACAAAAAGCTTAAAT, which was confirmed by DNA sequencing. This resulted in the C-terminal sequence of IEEAREQKA*, as originally engineered by Itakura et al. [8]. The 3’-terminal portion of the resultant S1∆IQ gene was subcloned into pTIKL GFP-L596S S1 to yield pTIKL GFP-L596S S1∆IQ.

GFP-S332D/G607A myoB-S1∆IQ: The *myoB* cDNA, provided by the National Bioresource Project, was modified by PCR-based procedures to engineer S332D and G607A mutations. The nucleotide and amino acid sequences around S332D are ATCcgtcgtGATacGtataac and NRRDTYN, respectively, where underlines indicate mutations. Those around the G607A mutation were CAAtatttggctcttctagag and QYLALLE, respectively. The nucleotide and amino acid sequences encompassing the 3’ end of the coding sequence was ttagaggaagcattggataagtaagagctc and LEEALDK*, where the underline shows the *Sac*I site used for subcloning into pDdNeo.

GFP-actin and mCherry-actin: GFP-actin was expressed using pBIG GFP-actin [9]. The sequence coding GFP moiety was replaced by that coding mCherry, and was inserted at the *BamH*I and *Sac*I sites of pDdBsr to express mCherry-actin.

References

1. Ruppel KM, Uyeda TQP, Spudich JA (1994) Role of highly conserved lysine 130 of myosin motor domain. J Biol Chem 269: 18773-18780.

2. Liu X, Ito K, Lee RJ, Uyeda TQP (2000) Involvement of tail domains in regulation of *Dictyostelium* myosin II. Biochem Biophys Res Commun 271: 75-81.

3. Dynes JL, Firtel RA (1989) Molecular complementation of a genetic marker in *Dictyostelium* using a genomic DNA library. Proc Natl Acad Sci USA 86: 7966-7970.

4. Yumura S, Uyeda TQP (1997) Transport of myosin II to the equatorial region without its own motor activity in mitotic *Dictyostelium* cells. Mol Biol Cell 8: 2089-2099.

5. Moores SL, Sabry JH, Spudich JA (1996) Myosin dynamics in live *Dictyostelium* cells. Proc Natl Acad Sci USA 93: 443-446.

6. Uyeda TQP, Patterson B, Mendoza L, Hiratsuka Y (2002) Amino acids 519-524 of *Dictyostelium* myosin II form a surface loop that aids actin binding by facilitating a conformational change. J Muscle Res Cell Motil 23: 685-695.

7. Patterson B, Ruppel KM, Wu Y, Spudich JA (1997) Cold-sensitive mutants G680V and G691C of *Dictyostelium* myosin II confer dramatically different biochemical defects. J Biol Chem 272: 27612-27617.

8. Itakura S, Yamakawa H, Toyoshima YY, Ishijima A, Kojima T, et al. (1993) Force-generating domain of myosin motor. Biochem Biophys Res Commun 196: 1504-1510.

9. Asano Y, Mizuno T, Kon T, Nagasaki A, Sutoh K, et al. (2004) Keratocyte-like locomotion in amiB-null *Dictyostelium* cells. Cell Motil Cytoskeleton 59: 17-27.
