# Supplementary material for: Stretching Actin Filaments within Cells Enhances their Affinity for the Myosin II Motor Domain
Source: PLoS One. 2011 Oct 13;6(10):e26200. doi: 10.1371/journal.pone.0026200 (PMC3192770; doi:10.1371/journal.pone.0026200)
Supplement: Text S2 — Comparison of the fluorescent probes for actin filaments. (DOC) [file pone.0026200.s014.doc]

Probes for actin filaments

Among the three distinct classes of probes to detect cellular actin filaments, i.e., GFP-actin [1,2,3], GFP-ABD of actin binding proteins, and rhodamine-phalloidin (Rh-Ph), Rh-Ph is well established as a general actin filament probe. However, Rh-Ph staining usually requires fixation of cells. Furthermore, cofilin was shown to inhibit Rh-Ph binding to actin filaments [4], potentially resulting in uneven staining of cellular actin filaments by Rh-Ph. Unlike Rh-Ph, GFP-based probes are easily applicable to live cell imaging. As a total actin filaments probe, GFP-actin is a straightforward choice, but monomeric pool also fluoresces, potentially making it difficult to detect weaker signals from filaments. Of the GFP-ABD class of actin filament probes, GFP-ABD of filamin (formerly ABP-120) was one of the first to be developed and was used to visualize actin filaments in live *Dictyostelium* cells [5]. However, Washington and Knecht [6] later demonstrated that this probe preferentially binds to cortical actin filaments, while binding only weakly to those in pseudopods, making it inappropriate to detect total actin filaments. More recently, GFP-Lifeact derived from ABD of ABP-140 of yeast was introduced as a versatile fluorescent probe for actin filaments [7]. Thus, GFP-Lifeact was expressed in wild type *Dictyostelium* cells, and after simultaneous permeabilization and fixation with Triton X-100 and glutaraldehyde, cells were stained with Rh-Ph, and observed under confocal fluorescence microscope. Superimposition of the two pseudocolored images of GFP and rhodamine showed that rhodamine fluorescence is relatively stronger along the front edge, whereas the GFP fluorescence was relatively stronger along the rear cortex in polarized cells (Supplemental Fig. S2A). This disagreement cannot be due to sensitivity of phalloidin staining to cofilin because cofilin is not enriched along the rear cortex in polarized cells [8], and is most likely due to preferential binding of Lifeact to actin filaments along the posterior cortex. Similarly, it was reported that Lifeact is unable to stain nuclear actin rods in mouse striated neuron-derived STHdh cells under stress [9]. In contrast to this, GFP-actin localization and Rh-Ph staining showed better agreement to one another after permeabilization and fixation (Supplemental Fig. S2B), confirming the previous report [2]. In this study, therefore, we decided to fix and permeabilize cells and stain them with Rh-Ph to semi-quantitatively visualize total actin filaments.

References

1. Doyle T, Botstein D (1996) Movement of yeast cortical actin cytoskeleton visualized in vivo. Proc Natl Acad Sci USA 93: 3886-3891.

2. Westphal M, Jungbluth A, Heidecker M, Muhlbauer B, Heizer C, et al. (1997) Microfilament dynamics during cell movement and chemotaxis monitored using a GFP-actin fusion protein. Curr Biol 7: 176-183.

3. Asano Y, Mizuno T, Kon T, Nagasaki A, Sutoh K, et al. (2004) Keratocyte-like locomotion in amiB-null *Dictyostelium* cells. Cell Motil Cytoskeleton 59: 17-27.

4. Hawkins M, Pope B, Maciver SK, Weeds AG (1993) Human actin depolymerizing factor mediates a pH-sensitive destruction of actin filaments. Biochemistry 32: 9985-9993.

5. Pang KM, Lee E, Knecht DA (1998) Use of a fusion protein between GFP and an actin-binding domain to visualize transient filamentous-actin structures. Curr Biol 8: 405-408.

6. Washington RW, Knecht DA (2008) Actin binding domains direct actin-binding proteins to different cytoskeletal locations. BMC Cell Biol 9: 10.

7. Riedl J, Crevenna AH, Kessenbrock K, Yu JH, Neukirchen D, et al. (2008) Lifeact: a versatile marker to visualize F-actin. Nat Methods 5: 605-607.

8. Aizawa H, Fukui Y, Yahara I (1997) Live dynamics of *Dictyostelium* cofilin suggests a role in remodeling actin latticework into bundles. J Cell Sci 110: 2333-2344.

9. Munsie LN, Caron N, Desmond CR, Truant R (2009) Lifeact cannot visualize some forms of stress-induced twisted F-actin. Nat Methods 6: 317.
